# Hospitalizations and mortality among patients with fetal alcohol spectrum disorders: a prospective study

**DOI:** 10.1038/s41598-020-76406-6

**Published:** 2020-11-11

**Authors:** Sarah Soyeon Oh, Young Ju Kim, Sung-in Jang, Sohee Park, Chung Mo Nam, Eun-Cheol Park

**Affiliations:** 1 Fetal Alcohol Syndrome Prevention Center, Ewha Womans University Mokdong Hospital, Seoul, Republic of Korea; 2 Institute of Health Services Research, Yonsei University College of Medicine, Seoul, Republic of Korea; 3 Department of Public Health & Preventive Medicine, Graduate School, Yonsei University College of Medicine, Seoul, Republic of Korea; 4 Department of Obstetrics & Gynecology, School of Medicine, Ewha Womans University, Seoul, Republic of Korea; 5 Department of Biostatistics, Yonsei University College of Medicine, Seoul, Republic of Korea

**Keywords:** Neuroscience, Physiology, Medical research, Neurology

## Abstract

With nearly 10% of women consuming alcohol during pregnancy, fetal alcohol spectrum disorders (FASDs) are becoming an increasing concern for clinicians and policymakers interested in the field of healthcare. Known as the range of mental and/or physical disabilities that occur among individuals with prenatal alcohol exposure, FASDs can result in dysmorphic features, problems with physical growth, neurobehavioral and cognitive problems that not only increase risk of various diseases, but also premature mortality. We investigated whether the diagnosis of FASDs result in increased risk of hospitalizations and mortality, with respect to FASD domains and relative diseases, when age effects are controlled for. The data for this study was taken from the National Health Insurance Service – National Sample Cohort (NHIS-NSC) between 2003 and 2013. The population attributable risk (PAR) statistic was used to estimate the percentage of hospitalizations and mortality attributable to FASDs and other factors. A time-dependent Cox proportional hazards model with age of diagnosis as the time-scale was employed to calculate adjusted hazard ratios and 95% CIs for hospitalizations and mortality among FASD populations compared to their general population peers. Among the 3,103 FASD cases, 27.5% experienced hospitalizations and 12.5% died. Overall, FASDs accounted for 853 FASD-attributable hospitalizations (51.0% of all hospitalizations in the study population) and 387 mortality events (34.5% of all deaths in the study population). 20.52% of hospitalizations and 21.35% of mortalities were attributable to FASDs in this population. Compared to the control group, FASD patients had a 1.25-fold (HR: 1.25, 95% CI: 1.05–1.49, p = 0.0114) increased risk of hospitalizations and a 1.33-fold (HR: 1.33, 95% CI: 1.07–1.67, p = 0.0118) increased risk of all-cause mortality. The most common cause for hospitalization was diseases of the nervous system, which accounted for 450 FASD-attributable hospitalizations (96.2% of all nervous system hospitalizations in the study population). In fact, FASD patients were 52 times more likely to be hospitalized for nervous system diseases than their peers (HR: 51.78, 95% CI: 29.09–92.17, p < .0001). The most common cause for mortality was neoplasms, which accounted for 94 FASD-attributable deaths (28.7% of all neoplasm deaths in the study population). However, FASD patients did not have increased risk of neoplasm mortality than the general population (HR: 0.88, 95% CI: 0.59–1.32, p < .0001). Overall, this study found that individuals diagnosed with FASDs have increased risk of both hospitalizations and mortality, compared to their general population peers. This is particularly so for diseases of the nervous system, which showed a 52-fold increase in hospitalizations and four-fold increase in mortality for FASD patients in our study. Likewise, while the association between FASDs and neoplasm mortality was not significant in our investigation, more attention by neurologists and related healthcare providers regarding the link between these two factors is necessary.

**Trial Registration:** Institutional Review Board of Yonsei University’s Health System: Y-2019-0174.

## Introduction

With reports that 9.8% of women consume alcohol during pregnancy, an increasing concern in the field of healthcare is the development of fetal alcohol spectrum disorders (FASD). FASDs are the range of mental and/or physical disabilities that can occur among all individuals with prenatal alcohol exposure. Systematically assessed according to various domains (dysmorphic features, physical growth, neurobehavioral development, and prenatal alcohol exposure)^1^, FASDs can result in a range of lifelong adverse outcomes, as well as increased risk for mortality^2,3^. Moreover, many problems that are associated with prenatal alcohol exposure are not currently considered on the FASD continuum, including isolated growth impairments, speech and language impairments, and vision impairments^4^. Such multimorbidity, deriving from prenatal alcohol exposure, is considered to be the leading preventable cause of congenital defects and mental impairment in the world^5^.

Unfortunately, the exact prevalence rate of FASDs is difficult to predict. Not only is alcohol consumption during pregnancy underreported, healthcare providers often misdiagnose or underdiagnose FASDs due to a lack of knowledge regarding diagnostic procedures and treatment recommendations^3,6^. Furthermore, while most cases of FASDs cannot be identified in infancy, no useful diagnostic criteria for FASD diagnosis among adults or the elderly exist^7^. Only the diagnostic criteria for infants, which is not predicative of FASD prevalence rates in childhood or adult life, allow us to estimate that FASDs occur among one in 13 prenatally exposed infants^6^.

Regionally, the prevalence estimate of alcohol use during pregnancy is highest in the European region (EUR) (PE: 25.2%, 95% CI: 21.6–29.6), followed by the region of the Americas (AMR) (PE: 11.2%, 95% CI: 9.4–12.6), African region (AFR) (PE: 10.0%, 95% CI: 8.5–11.8), Western-Pacific region (WPR) (PE: 8.6%, 95% CI: 4.5–11.6), South-East Asia region (PE: 1.8%, 95% CI: 0.9–5.1), and Eastern-Mediterranean region (EMR) (PE: 0.2%, 95% CI: 0.1–0.9)^8^. As such, the prevalence estimate of FASD by 10,000 persons are as follows: EUR (PE: 198.2, 95% CI: 140.9–280.0), AMR (PE: 87.9, 95% CI: 63.7–132.4), AFR (PE: 78.3, 95% CI: 53.6–107.1), WPR (PE: 67.4, 95% CI: 45.4–116.6), SEAR (PE: 14.1, 95% CI: 6.4–53.1), and EMR (PE: 1.3, 95% CI: 0.9–4.5)^9^. In the case of South Korea, previous studies have reported that around 13% to 16% of women are reported to drink during pregnancy^10^.

Current evaluations of the socio-economic costs of FASDs state that significant charges are also involved pertaining to healthcare, special education, residential care, the criminal justice system, productivity losses due to various comorbidities and diseases, premature mortality, and productivity losses due to caregiving of patients^11^.

As see in a systematic review of thirty-two studies from 4 countries (United States [n = 20], Canada [n = 9], Sweden [n = 2], and New Zealand [n = 1]), annual per-person costs of care can range from $22,810 per child to $24,308 per adult globally, which exceed costs for autism by 26%, asthma by 87%, diabetes by 13% and epilepsy by 56%^11^. Considering that around 630,000 new cases of FASDs are predicted to occur each year, this is a significantly costly problem warranting investments in prevention and treatment.

Although some studies have attempted to measure the complications associated with prenatal and primary care during pregnancy^12^, few recent studies have measured the prevalence and causes of hospitalizations in FASD population. What is known is that compared to their general population peers, children with FASDs are hospitalized more often for failure to thrive, neglect, anemia, child sexual abuse, and feeding problems and are often diagnosed with otitis media, pneumonia, dehydration, and anemia^13^.

Regarding premature mortality, previous studies have found that prenatal alcohol exposure is a powerful risk marker for spontaneous abortions, stillbirths, miscarriages, and deaths in general^14^. In a study comparing the mortality rates of siblings of children with FASDs and their general population peers, it was found that the siblings group’s mortality rate increased by 530%^15^. In another study comparing the mortality rates of subjects with FASDs and their same-age peers, cases with FASDs were at nearly five times the mortality risk for both children and adults^16^. Lastly, in a study of 175 human PNAE and FASD autopsy brains, Jarmasz et al. (2017) found placental abnormalities in at least 68% of fetal and infant cases, as well as various brain malformations including micrencephalies (17.8%), neural tube defects (3.4%), and minor leptomeningeal heterotopia (3.4%) among all subjects^17^.

Unfortunately, few studies have focused on the exact causes of mortality that occur among these groups in detail, especially in comparison to general population groups. Furthermore, types of FASDs pertaining to specific effects: fetal alcohol syndrome^18^, partial FAS (PFAS), static encephalopathy/alcohol exposed (SE/AE), and neurobehavioral disorder/alcohol exposed (ND/AE), and their respective associations with mortality have not been researched in-depth^19^.

Numerous comorbidities have been associated with prenatal alcohol exposure of the fetus and increased mortality risk. Conditions associated with the peripheral nervous system and special senses, conduct disorder, receptive language disorder, chronic serious otitis media, and expressive language disorders have shown to be especially prevalent with pooled estimates exceeding 50% of patients with FAS in certain studies (Table 1)^20^.

**Table 1:** ICD-10 Codes for FASDs (10th Revision)^55^.

Code	Description
P04.3	Newborn (suspected to be) affected by maternal use of alcohol (excludes FAS)
Q86.0	FAS (dysmorphic)—no further description offered for this specific code
F06.30	Mood disorder due to known physiological condition, unspecified
P00.4	Newborn (suspected to be) affected by maternal nutritional disorders
P01.9	Newborn (suspected to be) affected by maternal complication of pregnancy, unspecified
G93.4	Encephalopathy, other and unspecified (static)
G96.8	Other specified disorders of central nervous system
G96.9	Disorder of central nervous system, unspecified

Thus, medical expenditures that arise from such hospitalizations for children with FAS are noticeably higher than that of their general population peers. For example, a previous study reported that the annual mean medical expenditures of children with FAS are nine times higher than those of children without FASDs ($16,782 vs. $1,895)^21^. While most visits are outpatient visits in nature, FAS patients visit for inpatient services a lot more than their general population peers (31.3% vs. 17.2%)^21^.

Previous studies state that the cause-specific mortality rates of diseases on the FASD continuum contribute to around 6% of all deaths annually^22^. Causes range from external, including suicides and accidents, to internal, including diseases of the nervous and respiratory systems, digestive system, congenital malformations, mental and behavioral disorders, and diseases of the circulatory system^23^.

In other studies, malformations of the heart, brain malformations, including microcephaly, hydrocephaly, porencephaly etc.), sepsis, kidney malformations, and cancer were the most prevalent causes of death, especially among newborn in their first year of life, in which over half of all deaths occurred^24^.

Depending on early diagnosis and support, life expectancies can increase; however, on average, people with FAS are estimated to live 34 years (95% CI: 31–37 years), which is around 42% of the life expectancies of their general population peers^23^. Although initial diagnosis must be confirmed as early as possible to provide entry into intervention programs associated with improved outcomes, lack of an early diagnosis remains one of the strongest outcomes associated with disrupted school experience, trouble with the law, confinement in detention, jail^25^.

Thus, this study aimed to examine the mortality risk of patients with FASDs compared to their general population peers. Additionally, this study analyzed the effects of various sociodemographic characteristics on the impact of all-cause and cause-specific mortality, stratified by FASD domains.

## Materials and methods

### Data and study population

The data used in this study were obtained from the National Health Insurance Service National Sample Cohort (NHIS-NSC) for the years 2003 to 2013. This data was constructed with the sole purpose of providing public health researchers and policy makers with representative, useful information regarding Korean citizens’ utilization of health insurance and health examinations.

To construct the cohort, a representative sample cohort of 1,109,938 participants, representative of 2.2% of the Korean population, were randomly selected and followed for 11 years. During the follow-up period, a representative sample of newborns was added annually and all deceased or emigrated participants, excluded.

From these participants, we extracted 3,103 individuals who were classified as having FASDs by healthcare professionals from the database, using the ICD-10 codes for FASDs, standardized by the American Academy of Pediatrics.

**Figure 1:**
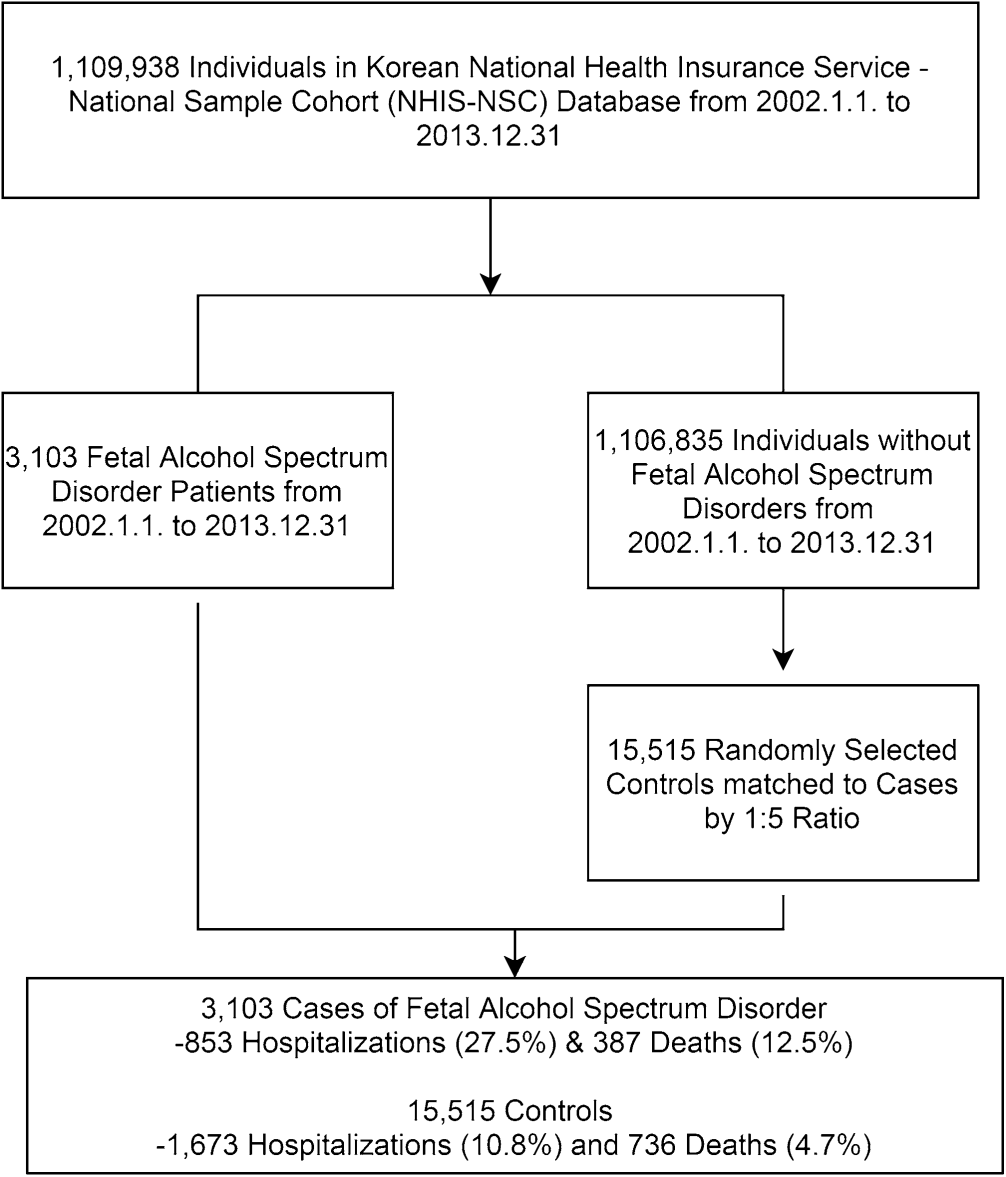
Flow chart of the study population.

While the ICD-10 diagnoses was employed in our study, it must be noted that the use of electronic health records (EHRs) in FASD diagnoses has numerous problems including the lack of a specific code or gold standard for the identification of FAS, and the reticence of physicians to make the diagnosis of FASDs due to the various social stigmas it may imply and/or difficulties in identification of characteristics^26^. Controls were selected randomly from the 1,106,835 individuals without a diagnosis of FASDs in our study period (Fig. 1).

### Measures

The dependent variables of this study were hospitalizations and all-cause mortality, as well as type of hospitalization and mortality from other causes. Only hospitalizations and/or mortality cases occurring within the study period, following the time-dependent exposure of FASD diagnosis were seen as events. Hospitalization and death codes were classified according to ICD-10 chapters, excluding codes for FASDs, as done so in the existing body of literature^27^. The independent variable in this study was the diagnosis of FASDs, compared to general population peers. In our analysis, the diagnosis of FASDs was a time-dependent covariate; resulting in the censoring of any event occurring before the diagnosis of FASDs to prevent time-dependent bias.

Covariates controlled for while investigating the association between prevalence of FASDs and mortality included sex, income, medical insurance, disability, disability severity, disability type, level of psychatric treatment, Charlson Comorbidity Index Score^28^, region, and year of cohort entry.

### Statistical analysis

Chi-squared tests and univariate analysis were conducted to examine the distribution of the general characteristics of the study population, with regard to cases, controls, and the total population. General characteristics were reported through frequencies and percentages. The population attributable risk (PAR) statistic was used to estimate the percentage of hospitalizations and mortality that could be attributed to FASDs and other factors in this population. This was calculated on the basis of the following formula: PAR = (p[r − 1]/(p[r − 1] + 1) × 100.

To calculate the association between FASD diagnosis and the risks of hospitalizations and mortality, a time-dependent Cox’s proportional hazards regression model was employed to calculate adjusted hazard ratio (HR) and 95% CI (confidence interval) values.

While the most common time scale for Cox’s proportional hazards analyses is follow-up period with adjusted baseline entry age, this study employed attained age as the time scale with left truncated data. Many studies have recently argued that in time-on studies where follow-up period is tracked from cohort entry to a certain event (e.g. death), the effect of age is not tightly controlled for^29^. For example, if follow-up period is employed as the time scale, a child patient may have the same time scale as a middle-aged or elderly patient, who was admitted to the cohort for the same length of time. By employing age as our time scale, our study was able to calculate age-specific incidence functions, with flexible control for the effects of age.

In our study, age at recruitment of study was defined as the first admission into our cohort for both cases and controls. Because the NHIS-NSC categorizes the ages of individuals into groups of 5, median age was used to calculate entry age. For all values calculated in our study, a two-tailed *p* value of < 0.05 was considered statistically significant. All statistical analyses were conducted using SAS (ver. 9.4).

### Statement

All methods were performed in accordance with relevant guidelines and regulations. All experimental protocols were approved by the Institutional Review Board of Yonsei University’s Health System (Y-2019–0174). The need for informed consent was waived since the NHIS-NSC provides anonymous cohort data to researchers for scholarly use.

## Results

Table 2 shows the general characteristics and distribution of the FASD case group and matched controls. The case group included 3,103 individuals, while the control group included 15,515 individuals. The case group consisted of 16.7% of the study population while the control group consisted of 83.3% of the study population. Chi-square test values were used to compare the FASD case group to the control group.

**Table 2:** General characteristics of study population with FASD and general population.

Variables	Total		Fetal Alcohol Spectrum Disorder		General Population		*p*-value
	n	%	n	%	n	%	
**Sex**							
Male	9057	48.6	1364	44.0	7693	49.6	< .0001
Female	9561	51.4	1739	56.0	7822	50.4	
**Age at Baseline**							
< 20	5565	29.9	313	10.1	5252	33.9	< .0001
20–29	2672	14.4	265	8.5	2407	15.5	
30–39	3091	16.6	399	12.9	2692	17.4	
40–49	2838	15.2	599	19.3	2239	14.4	
50–59	1819	9.8	556	17.9	1263	8.1	
60–69	1490	8.0	551	17.8	939	6.1	
≥ 70	1143	6.1	420	13.5	723	4.7	
**Income**							
None	513	2.8	114	3.7	399	2.6	
Low	2140	11.5	366	11.8	1774	11.4	< .0001
Medium–low	2806	15.1	451	14.5	2355	15.2	
Medium	3640	19.6	541	17.4	3099	20.0	
Medium–high	4496	24.1	695	22.4	3801	24.5	
High	5023	27.0	936	30.2	4087	26.3	
**Medical Insurance**							
Medical Aid	513	2.8	114	3.7	399	2.6	
Insurance (Regional)	8632	46.4	1428	46.0	7204	46.4	0.0028
Insurance (Corporate)	9473	50.9	1561	50.3	7912	51.0	
**Disability Type**							
None	17,865	96.0	2783	89.7	15,082	97.2	< .0001
Mental	492	2.6	219	7.1	273	1.8	
Sensory	122	0.7	38	1.2	84	0.5	
Intellectual	69	0.4	28	0.9	41	0.3	
Other*	70	0.4	35	1.1	35	0.2	< .0001
**Disability Severity**							
No Disability	17,865	96.0	2783	89.7	15,082	97.2	
Level 1–2	301	1.6	156	5.0	145	0.9	
Level 3–6	452	2.4	164	5.3	288	1.9	
**Charlson Comorbidity Index**							
None	4547	24.4	249	8.0	4298	27.7	< .0001
One	4649	25.0	367	11.8	4282	27.6	
Two	2465	13.2	339	10.9	2126	13.7	
≥ Three	6957	37.4	2,148	69.2	4809	31.0	
**Region**							
Metropolitan	8123	43.6	1,583	51.0	6540	42.2	< .0001
City	4730	25.4	601	19.4	4129	26.6	
Other	5765	31.0	919	29.6	4846	31.2	
**Year of Cohort Entry**							
2003	11,398	61.2	172	5.5	11,226	72.4	< .0001
2004	1740	9.3	180	5.8	1560	10.1	
2005	942	5.1	192	6.2	750	4.8	
2006	676	3.6	255	8.2	421	2.7	
2007	589	3.2	287	9.2	302	1.9	
2008	715	3.8	333	10.7	382	2.5	
2009	497	2.7	283	9.1	214	1.4	
2010	529	2.8	339	10.9	190	1.2	
2011	583	3.1	397	12.8	186	1.2	
2012	546	2.9	386	12.4	160	1.0	
2013	403	2.2	279	9.0	124	0.8	
Total	18,618	100.0	3103	100.0	15,515	100.0	

*Kidney, musculoskeletal, circulatory, respiratory, nervous system, developmental etc.

Table 3 shows the general characteristics and distribution of hospitalizations and all-cause mortality, attained through univariate analysis. Among the 3,103 FASD cases, 27.5% (n = 853) experienced hospitalizations and 12.5% (n = 387) died. 20.52% of hospitalizations and 21.35% of mortalities were attributable to FASDs in this population.

**Table 3:** General Characteristics of Study Population by Hospitalizations and All-Cause Mortality.

	**Hospitalization**									**All-Cause Mortality**								
	**Yes**		**No**		**RR**	**95% CI**			**PAR %**	**Yes**		**No**		**RR**	**95% CI**			**PAR %**
	**n**	**%**	**n**	**%**		**Lower**		**Upper**		**n**	**%**	**n**	**%**		**Lower**		**Upper**	
**Fetal Alcohol Spectrum Disorder**																		
None	1,673	10.80	13,842	89.2	1.00	–		–		736	4.7	14,779	95.3	1.00	–		–	
Fetal Alcohol Spectrum Disorder	853	27.50	2,250	72.5	2.55	2.37	–	2.74	20.52	387	12.5	2,716	87.5	2.63	2.63	–	2.63	21.35
**Sex**																		
Male	1,197	13.20	7,860	86.8	1.00	–		–		616	6.8	8,441	93.2	1.00	–		–	
Female	1,329	13.90	8,232	86.1	1.05	0.98	–	1.13	2.59	507	5.3	9,054	94.7	0.78	0.78	–	0.78	–12.76
**Age at Baseline**																		
< 20	424	7.60	5,141	92.4	1.00	–		–		8	0.1	5,557	99.9	1.00	–		–	
20–29	257	9.60	2,415	90.4	1.26	1.09	–	1.46	7.84	12	0.4	2,660	99.6	3.12	3.12	–	3.12	40.79
30–39	336	10.90	2,755	89.1	1.43	1.25	–	1.63	13.22	27	0.9	3,064	99.1	6.08	6.08	–	6.08	64.45
40–49	414	14.60	2,424	85.4	1.91	1.69	–	2.18	23.60	72	2.5	2,766	97.5	17.65	8.51	–	36.59	84.90
50–59	320	17.60	1,499	82.4	2.31	2.02	–	2.64	24.38	125	6.9	1,694	93.1	47.80	23.44	–	97.51	92.02
60–69	370	24.80	1,120	75.2	3.26	2.87	–	3.70	32.30	212	14.2	1,278	85.8	98.97	48.97	–	200.03	95.39
≥ 70	405	35.40	738	64.6	4.65	4.12	–	5.25	38.35	667	58.4	476	41.6	405.93	202.76	–	812.71	98.57
**Income**																		
None	115	22.40	398	77.6	1.00	–		–		111	21.6	402	78.4	1.00	–		–	
Low	320	14.95	1,820	85	1.25	1.04	–	1.51	14.81	138	6.4	2,002	93.6	0.30	0.24	–	0.38	–130.54
Medium–low	392	13.97	2,414	86	2.17	1.82	–	2.59	41.68	147	5.2	2,659	94.8	0.24	0.19	–	0.30	–178.35
Medium	455	12.50	3,185	87.5	0.56	0.46	–	0.67	–63.33	173	4.8	3,467	95.2	0.22	0.18	–	0.27	–216.41
Medium–high	575	12.79	3,921	87.2	0.57	0.48	–	0.68	–62.74	199	4.4	4,297	95.6	0.20	0.17	–	0.25	–249.62
High	669	13.32	4,354	86.7	0.59	0.50	–	0.71	–58.29	355	7.1	4,668	92.9	0.33	0.27	–	0.40	–157.05
**Medical Insurance**																		
Medical Aid	115	22.40	398	77.6	1.74	1.47	–	2.07	3.68	111	21.6	402	78.4	3.31	2.76	–	3.96	10.59
Insurance (Regional)	1,193	13.80	7,439	86.2	1.07	1.00	–	1.16	3.45	392	4.5	8,240	95.5	0.69	0.61	–	0.78	–17.09
Insurance (Corporate)	1,218	12.90	8,255	87.1	1.00	–		–		620	6.5	8,853	93.5	1.00	–		–	
**Disability Type**																		
None	2,266	12.70	15,599	87.3	1.00	–		–		828	4.6	17,037	95.4	1.00	–		–	
Mental	171	34.80	321	65.2	2.74	2.41	–	3.11	4.46	184	37.4	308	62.6	8.07	7.07	–	9.21	15.93
Sensory	36	29.50	86	70.5	2.33	1.76	–	3.07	0.89	45	36.9	77	63.1	7.96	6.25	–	10.13	4.51
Intellectual	19	27.50	50	72.5	2.17	1.48	–	3.19	0.45	12	17.4	57	82.6	3.75	2.23	–	6.30	1.05
Other*	34	48.60	36	51.4	3.83	3.00	–	4.89	1.09	54	77.1	16	22.9	16.64	14.41	–	19.22	5.75
**Disability Severity**																		
No Disability	2,266	12.70	15,599	87.3	1.00	–		–		828	4.6	17,037	95.4	1.00	–		–	
Level 1–2	120	39.90	181	60.1	3.14	2.72	–	3.63	3.43	150	49.8	151	50.2	10.75	9.43	–	12.26	13.91
Level 3–6	140	31.00	312	69	2.44	2.12	–	2.82	3.44	145	32.1	307	67.9	6.92	5.96	–	8.04	12.75
**Charlson Comorbidity Index**																		
None	280	6.20	4,267	93.8	1.00	–		–		116	2.6	4,431	97.4	1.00	–		–	
One	412	8.90	4,237	91.1	1.44	1.24	–	1.67	18.17	111	2.4	4,538	97.6	0.94	0.72	–	1.21	-3.35
Two	299	12.10	2,166	87.9	1.97	1.69	–	2.30	25.42	135	5.5	2,330	94.5	2.15	1.68	–	2.74	28.73
≥ Three	1,535	22.10	5,422	77.9	3.58	3.17	–	4.05	60.97	761	10.9	6,196	89.1	4.29	3.54	–	5.19	66.54
**Region**																		
Metropolitan	764	9.40	7,359	90.6	1.00	–		–		440	5.4	7,683	94.6	1.00	–		–	
City	609	12.90	4,121	87.1	1.37	1.24	–	1.51	11.95	249	5.3	4,481	94.7	0.97	0.84	–	1.13	–1.05
Other	1,153	20.00	4,612	80	2.13	1.95	–	2.32	0.04	434	7.5	5,331	92.5	1.39	1.22	–	1.58	13.93
**Year of Cohort Entry**																		
2003	1,276	11.20	10,122	88.8	1.00	–		–		632	5.5	10,766	94.5	1.00	–		–	
2004	196	11.30	1,544	88.7	1.01	0.87	–	1.16	0.08	83	4.8	1,657	95.2	0.86	0.69	–	1.08	–1.87
2005	124	13.20	818	86.8	1.18	0.99	–	1.40	1.32	59	6.3	883	93.7	1.13	0.87	–	1.46	0.99
2006	85	12.60	591	87.4	1.12	0.91	–	1.38	0.68	56	8.3	620	91.7	1.08	0.83	–	1.40	0.58
2007	117	19.90	472	80.1	1.77	1.50	–	2.10	3.67	50	8.5	539	91.5	1.53	1.16	–	2.02	2.55
2008	147	20.60	568	79.4	1.84	1.58	–	2.14	4.71	87	12.2	628	87.8	2.20	1.78	–	2.71	6.59
2009	95	19.10	402	80.9	1.71	1.41	–	2.06	2.87	38	7.6	459	92.4	1.38	1.01	–	1.89	1.56
2010	115	21.70	414	78.3	1.94	1.64	–	2.30	4.01	43	8.1	486	91.9	1.47	1.09	–	1.97	2.03
2011	145	24.90	438	75.1	2.22	1.91	–	2.58	5.61	35	6	548	94	1.08	0.78	–	1.51	0.41
2012	128	23.40	418	76.6	2.09	1.78	–	2.46	4.76	28	5.1	518	94.9	0.93	0.64	–	1.34	–0.34
2013	98	24.30	305	75.7	2.17	1.81	–	2.60	3.85	12	3	391	97	0.54	0.31	–	0.94	–1.60
**Total**	2,526	13.60	16,092	86.4						1,123	6	17,495	94					

*Kidney, musculoskeletal, circulatory, respiratory, nervous system, developmental etc.

Among the control group, 10.8% (n = 1,673) experienced hospitalizations and 4.7% (736) died. Overall, 13.6% of our study population experienced some form of hospitalization during the study period, while 6.0% died from various causes.

Table 4 shows the results of the Cox proportional hazards analysis of the FASD group and controls with attained age as the underlying timescale, while adjusting for covariates. Compared to the control group, FASD cases had increased risk of hospitalizations (HR: 1.25, 95% CI: 1.05–1.49, p = 0.0114) and all-cause mortality (HR: 1.33, 95% CI: 1.07–1.67, p = 0.0118). Regarding hospitalizations, compared to individuals with corporate insurance coverage, individuals with regional insurance coverage had slightly increased risk of hospitalization (HR: 1.12, 95% CI: 1.02–1.22, p = 0.0157).

**Table 4:** Results of Cox Model for FASD and Hospitalizations/Mortality with Attained Age as Underlying Timescale.

	Hospitalization					All-Cause Mortality				
	HR	95% CI			p-value	HR	95% CI			p-value
		Lower		Upper			Lower		Upper	
**Fetal Alcohol Spectrum Disorder**										
None	1.00	–		–		1.00	–		–	
Fetal Alcohol Spectrum Disorder	1.25	1.05	–	1.49	0.0114	1.33	1.07	–	1.67	0.0118
**Sex**										
Male	1.00	–		–		1.00	–		–	
Female	1.01	0.93	–	1.11	0.7905	0.60	0.52	–	0.68	< .0001
**Income**										
None	1.15	0.86	–	1.53	0.3392	1.57	1.15	–	2.12	0.0040
Low	1.01	0.86	–	1.19	0.8948	0.68	0.53	–	0.88	0.0033
Medium–low	1.08	0.93	–	1.26	0.3094	0.84	0.66	–	1.08	0.1722
Medium	1.00	–		–		1.00	–		–	
Medium–high	1.04	0.91	–	1.20	0.5428	0.73	0.58	–	0.92	0.0066
High	1.00	0.88	–	1.15	0.9822	0.72	0.58	–	0.88	0.0018
**Medical Insurance**										
Medical Aid	–					–				
Insurance (Regional)	1.12	1.02	–	1.22	0.0157	0.94	0.81	–	1.09	0.3999
Insurance (Corporate)	1.00	–		–		1.00	–		–	
**Disability Type**										
None	1.00	–		–		1.00	–		–	
Mental	1.44	1.14	–	1.82	0.0020	1.93	1.53	–	2.42	< .0001
Sensory	1.23	0.81	–	1.86	0.3322	1.15	0.80	–	1.65	0.4668
Intellectual	2.20	1.23	–	3.95	0.0081	3.77	1.85	–	7.69	0.0003
Other*	2.19	1.35	–	3.56	0.0016	2.63	1.84	–	3.75	< .0001
**Disability Severity**										
No Disability	1.00	–		–		1.00	–		–	
Level 1–2	1.24	0.91	–	1.70	0.1797	1.81	1.39	–	2.36	< .0001
Level 3–6	–					–				
**Charlson Comorbidity Index**										
None	1.00	–		–		1.00	–		–	
One	1.42	1.19	–	1.68	< .0001	0.69	0.50	–	0.94	0.0197
Two	1.84	1.53	–	2.20	< .0001	0.84	0.63	–	1.13	0.2552
≥ Three	2.75	2.36	–	3.21	< .0001	0.73	0.57	–	0.94	0.0145
**Region**										
Metropolitan	1.00	–		–		1.00	–		–	
City	1.58	1.40	–	1.78	< .0001	1.01	0.85	–	1.21	0.9054
Other	2.41	2.17	–	2.68	< .0001	1.02	0.87	–	1.18	0.8263
**Year of Cohort Entry**										
2003	1.00	–		–		1.00	–		–	
2004	1.57	1.33	–	1.84	< .0001	1.23	0.95	–	1.59	0.1236
2005	2.52	2.05	–	3.11	< .0001	1.43	1.04	–	1.97	0.0259
2006	2.94	2.23	–	3.86	< .0001	1.62	1.12	–	2.35	0.0105
2007	5.97	4.55	–	7.82	< .0001	1.53	1.05	–	2.21	0.0265
2008	7.77	5.95	–	10.16	< .0001	2.11	1.53	–	2.92	< .0001
2009	8.33	5.96	–	11.64	< .0001	2.35	1.54	–	3.57	< .0001
2010	16.72	12.09	–	23.12	< .0001	2.62	1.71	–	4.03	< .0001
2011	19.84	13.60	–	28.93	< .0001	1.86	1.12	–	3.09	0.0171
2012	16.42	9.26	–	29.13	< .0001	2.70	1.35	–	5.39	0.0050
2013	–		–			–				

*Kidney, musculoskeletal, circulatory, respiratory, nervous system, developmental etc.

Table 5 shows the results of the subgroup analyses for the impact of FASDs on various cause-specific hospitalizations while adjusting for sex, income, medical insurance, disability, disability severity, disability type, Charlson Comorbidity Index Score^28^, region, and year of cohort diagnosis. Of the 2,526 ever hospitalizations that occurred within our study period, 27.5% of FASD patients and 10.8% of the general population controls experienced hospitalizations. The most common cause for hospitalization was diseases of the nervous system, which FASD patients showed a 52-fold increase for hospitalizations than their peers (HR: 51.78, 95% CI: 29.09–92.17, p < 0.0001).

**Table 5:** Results of Subgroup Analysis on Impact of FASDs on Hospitalization, stratified by Hospitalization Cause.

	FASD (n = 3,103)		General Population (n = 15,515)		None	Hospitalization				
						HR	95% CI			p-value
	n	%	n	%			Lower		Upper	
**Hospitalization Causes**										
**All-Causes**	**853**	**27.5**	**1,673**	**10.8**	**1.00**	**1.25**	**1.05**	–	**1.49**	**0.0114**
Nervous	450	14.5	18	0.1	1.00	51.78	29.09	–	92.17	< .0001
Mental	179	5.8	32	0.1	1.00	7.87	4.34	–	14.26	< .0001
Circulatory	59	1.9	112	0.5	1.00	1.18	0.67	–	2.09	0.5748
Abnormal	32	1.0	26	0.1	1.00	1.97	0.72	–	5.38	0.1861
Musculoskeletal	31	1.0	138	0.6	1.00	0.33	0.16	–	0.68	0.0027
Digestive	23	0.7	135	0.6	1.00	0.30	0.14	–	0.63	0.0014
Neoplasm	22	0.7	19	0.1	1.00	0.94	0.27	–	3.23	0.9230
Eye and Ear	17	0.5	158	0.7	1.00	0.41	0.19	–	0.88	0.0227
Endocrine	11	0.4	35	0.2	1.00	0.41	0.11	–	1.55	0.1878
Other*	29	0.9	1,000	.4	1.00	0.08	0.05	–	0.14	< .0001

*Infectious, genitourinary, respiratory, external, perinatal, congenital, respiratory, skin.

Table 6 shows the results of the subgroup analyses for the impact of FASDs on various cause-specific mortality cases. Of the 1,123 deaths that occurred within our study period, 12.5% of FASD patients and 4.7% of the general population controls experienced death. The most common cause for mortality was neoplasms although FASD patients did not have increased risk of neoplasm mortality than the general population (HR: 0.88, 95% CI: 0.59–1.32, p < 0.0001). FASD patients had greatest risk of mortality from diseases of the nervous system (HR: 4.15, 95% CI: 1.54–11.17, p = 0.0049) than their peers. Compared to their general population peers, FASD cases also had increased risk of mortality from circulatory (HR: 2.07, 95% CI: 1.34–3.18, p < 0.0001), digestive (HR: 3.63, 95% CI: 1.66–7.93, p = 0.0012), respiratory (HR: 2.51, 95% CI: 1.15–5.48, p = 0.0215), and endocrine (HR: 3.04, 95% CI: 1.33–6.96, p = 0.0085) diseases.

**Table 6:** Results of Subgroup Analysis on Impact of FASDs on Mortality, stratified by Mortality Cause.

	FASD (n = 3,103)		General Population (n = 15,515)		None	Mortality				
						HR	95% CI			p-value
	n	%	n	%			Lower		Upper	
**Mortality Causes**										
**All-Causes**	**387**	**12.5**	**736**	**4.7**	**1.00**	**1.33**	**1.07**	–	**1.67**	**0.0118**
Neoplasm	94	3.0	234	1.5	1.00	0.88	0.59	–	1.32	< .0001
Circulatory	89	2.9	151	1.0	1.00	2.07	1.34	–	3.18	< .0001
Digestive	35	1.1	30	0.2	1.00	3.63	1.66	–	7.93	0.0012
Respiratory	31	1.0	36	0.2	1.00	2.51	1.15	–	5.48	0.0215
Endocrine	29	0.9	33	0.2	1.00	3.04	1.33	–	6.96	0.0085
Nervous	26	0.8	19	0.1	1.00	4.15	1.54	–	11.17	0.0049
External*	25	0.8	94	0.6	1.00	1.26	0.65	–	2.44	0.4957
Abnormal	20	0.6	73	0.5	1.00	0.77	0.32	–	1.85	0.5590
Infectious	17	0.5	22	0.1	1.00	2.22	0.72	–	6.81	0.1647
Other**	21	0.7	44	0.3	1.00	0.77	0.72	–	0.82	< .0001

*Suicides, accidents, poisoning by illegal drugs or alcohol.

**Pregnancy, mental, musculoskeletal, missing.

Table 7 shows the results of the subgroup analyses for the impact of the various types of FASDs on all-cause mortality. Compared to the control group, FASD cases diagnosed with ‘F06.30′ i.e. mood disorder due to known physiological condition, unspecified (HR: 1.30, 95% CI: 1.03–1.65, p = 0.0308), ‘G96.8′ i.e. other specified disorders of the central nervous system (HR: 2.15, 95% CI: 1.29–3.59, p = 0.0035), and ‘G96.9′ i.e. disorder of central nervous system, unspecified (HR: 1.77, 95% CI: 1.30–2.40, p = 0.0002) had increased hospitalization risk. Compared to the control group, FASD cases diagnosed with ‘G93.4′ i.e. encephalopathy, other and unspecified (static) (HR: 1.49, 95% CI: 1.17–1.91, p = 0.0014) and ‘G96.8′ i.e. other specified disorders of the central nervous system (HR: 2.04, 95% CI: 1.03–4.07, p = 0.0422) had increased mortality risk.

**Table 7:** Results of Subgroup Analyses on Type of FASD and Hospitalizations/Mortality.

	FASD		Hospitalization						All-Cause Mortality				
			None	HR	95% CI			p-value	HR	95% CI			p-value
	n	%			Lower		Upper			**Lower**		**Upper**	
**Type of FASD**													
**All Cases**	3,103	100.0	1.00	1.25	1.05	–	1.49	0.0114	1.33	1.07	–	1.67	0.0118
P04.3	1	0.0	1.00	–					–				
Q86.0	0	0.0	1.00	–					–				
F06.30	781	25.2	1.00	1.30	1.03	–	1.65	0.0308	1.17	0.87	–	1.57	0.3066
P00.4	0	0.0	1.00	–					–				
P01.9	3	0.1	1.00	–					–				
G93.4	2,015	64.9	1.00	1.10	0.91	–	1.33	0.3374	1.49	1.17	–	1.91	0.0014
G96.8	79	2.5	1.00	2.15	1.29	–	3.59	0.0035	2.04	1.03	–	4.07	0.0422
G96.9	224	7.2	1.00	1.77	1.30	–	2.40	0.0002	0.89	0.52	–	1.50	0.6485

*P04.3 Newborn (suspected to be) affected by maternal use of alcohol (excludes FAS).

Q86.0 FAS (dysmorphic)—no further description offered for this specific code.

F06.30 Mood disorder due to known physiological condition, unspecified.

P00.4 Newborn (suspected to be) affected by maternal nutritional disorders.

P01.9 Newborn (suspected to be) affected by maternal complication of pregnancy, unspecified.

G93.4 Encephalopathy, other and unspecified (static).

G96.8 Other specified disorders of central nervous system.

G96.9 Disorder of central nervous system, unspecified.

## Discussion

Ultimately, the aim of our investigation was to investigate the impact of FASD diagnosis on hospitalizations and mortality compared to the general population. A major strenght of our investigation is that we were able to reference data from the NHIS claims database for the years 2003 and 2013, making our data representative of approximately 2% of the Korean population. Few studies have employed a dataset of this size to investigate the effects of FASD diagnosis on individuals’ health statuse. Furthermore, we were able to gather data for medical treatment records with diagnosis codes, giving us the opportunity to analyze the morbidity and mortality prevalence and hazard ratios of individuals cause-specifically (via ICD-10 codes implemented in electronic health records by healthcare professionals).

Because such few studies have attempted to investigate FASDs in South Korea and other countries, our analysis is unique in that prevalence of diagnosis, as well as cause-specific and all-cause hospitalizations and mortality could be calculated for. However, a number of limitations must be noted. Age is only presented in the NHIS-NSC in five-year intervals; thus, we had to employ a median value for the entry and exit age of all subjects. It is highly recommended that future studies employing attained age as the time scale employ data with exact age of cohort entry, cohort exit, and diagnosis.

While the concept of hospitalizations has been employed in many previous studies, more studies are required to investigate how FASD patients differ from the general population with regard to hospitalization-reated characteristics like length of stay, medical costs etc. to further this investigation.

In our study, insurance claims were the only source of information to gather a large sample size for FASDs, however, severity of the disease, social factors, patients’concerns and expectations are all factors that are impossible to control for in such situations^30^. Rashidian and colleagues have emphasized that while ICD codes are imputed by professionals who are trained and certified, there is substantial variability in human coding, as well as the identification and documentation of disease by different clinicians^31^. In the case of neurobehavioral disorders associated with prenatal alcohol exposure like fetal alcohol spectrum disorders, the only relevant terms to conditions within FASDs, as used in our investigation, are “fetal alcohol syndrome, dysmorphic” (Q86.0), and “newborn (suspected to be) affected by maternal use of alcohol” (P04.3), which are limited in describing the lifelong symptoms of PAE relative to FASDs.

Moreover, FASDs in nature have been difficult to diagnose as they co-occur or have similar symptoms to other attentional, neurocognitive, and/or adaptive function deficit disorders like attention-deficit/hyperactivity disorder (ADHD) or Down’s Syndrome^32^. Certain symptoms have been likened to mood problems found in individuals with bipolar disorder and depressive disorders, and require ongoing identification of prenatal alcohol exposure, substance abuse or dependence, growth delay and central nervous system anomalies to distinguish from other pathologies^33^.

Likewise, while most cases of FASDs cannot be identified in infancy, no useful criteria for adults or the elderly have currently been validified. As highlighted by Burd (2016), we need to figure out how to identify FASDs in adults and the elderly, especially because FASD symptoms among the elderly do not seem to resemble that of children and adolescents^7^.

Additionally, while there are ICD-10 codes for FAS, no exact code exists for FASDs, and requires the researcher to estimate the number of FASD cases relative to the diagnostic code fields given. As previously mentioned in our literature review, records that can be reviewed for prenatal alcohol exposure range from newborn history forms, admission history and physical examinations, progress notes and discharge physical examinations, to obstetrical records of the obstetric nurses’ labor and delivery admission checklist and progress notes^34^. However, there are substantial limitations to recording notations regarding the use of alcohol, cigarettes, recreational drugs, and/or harmful medications as ICD-10 codes for FAS are limited^34^.

Because of the nature of the dataset, we were unable to control for certain confounding characteristics that may have been associated with FASD diagnosis and mortality, such as the drinking behaviors of the mother or father of the patient, or knowledge of prenatal alcohol exposure. Had details regarding drinking frequency and/or amount during pregnancy been controlled for, associations between exposure to lesser amounts of alcohol and hospitalizations and mortality could have been investigated.

Previous studies have found that exposure to smaller amounts of alcohol are associated with significant fetal effects including reduction in birth weight, birth length, and head circumference; the presence of the typical alcohol-related craniofacial features; and significant cognitive and behavioral manifestations^34^. Likewise, prenatal alcohol exposure, even in small amounts, has been associated with cranofacial development problems; especially among mothers who feel the effects of alcohol quickly, and who drink throughout pregnancy^35^. Cranofacial abnormalities ranging from an elevation of the lower portion of the nose, smooting of the philtrum, reduced palpebral fissure length, midfacial hypoplasia, and retrognathia, have been strongly prevalent among infants of mothers who drank moderately during their first trimester of pregnancy^35^.

Although disability severity and type were controlled for, we were unable to obtain exact information regarding the severity of FASDs in patients’ lives with regard to primary and secondary disabilities.

We were also unable to control for the exact date of diagnosis, which warrants future research as previous studies have acknowledged a strong association between early diagnosis of FASDs and fewer primary and/or secondary disabilities such as alcohol/drug problems, trouble with the law, and/or confinement^36^. Fetal alcohol biomarkers and genetic research are believed to play a central role in determining the onset of FASDs, however, due to the nature of our dataset, we were unable to control for related variables. Considerably more research regarding this area is warranted^37^.

Finally, while controlling for age was the main aim of our research design, it was impossible to control for the proportion of mortality events that occurred before the case could be identified. As noted in previous studies, prenatal alcohol exposure is associated with increased risk of various birth outcomes, including stillbirths, miscarriage, and sudden infant death syndrome^38,39^. Because our sample was obtained through ICD-10 diagnoses that were obtained throughout over 10 years of follow up, some forms of survival bias will exist and this should be considered when interpreting results.

Despite these limitations, this study was able to demonstrate that compared to their general population peers, individuals with FASDs have increased risk of both hospitalizations and mortality, even when age effects are controlled for. As seen in Supplementary Tables 1 and 2, both hospitalizations and mortality among individuals with FASDs were affected by age effects, which resulted in different hazard ratios between the two study designs.

With our subgroup analyses, we were also able to see that men with FASDs have increased risk of hospitalization compared to men without FASDs, and women with FASDs have increased risk of mortality compared to women without FASDs. Similarly, those in medium–high income groups have increased risk of hospitalizations compared to their general population peers when diagnosed with FASDs, as do those with no disability diagnosis. Finally, individuals with one comorbidity may have increased risk of mortality compared to the general population, as well as those living in regions that are not in the city or metropolitan regions.

Furthermore, because of its population-based design and decade-long follow up, the study population is nationally representative. The NHIS-NSC database contains nationally representative population-based data on more than one million participants, generated by the government so that researchers and policymakers can access extensive information regarding electroic medical treatment bills, bill details, details of diseases, details of prescriptions, details of birth, and deaths^40^. Selection bias is limited as the primary sampling unit is the individual and demographic characteristics are not lost as the sampling strategy is cohort based.

Through the use of a left truncated cohort with attained age as the time scale, we were able to avoid the biases that occur in time-on studies. Although starting points are relatively easy to identify for clinical studies, in epidemiological studies where the beginning of risk-periods are difficult to determine, time-scales may be warranted. Thiebaut and colleagues “strongly recommend not using time-on-study as the time scale for analysing epidemiologic cohort data”, especially when covariates of interest are either time-dependent or strongly associated with age^41^. Since both these cases are true in our investigation and there is a substantial association between age and our covariate under study (FASD diagnosis), it was important to address these biases in our model.

Lastly, we employed objective indicators including the hospitalization index, which scholars have confirmed to be an indicator of primary care quality, when appropriate adjustment factors are applied for^28^.

With regard to hospitalizations, we found that FASD diagnosis is associated with increased risk of hospitalizations, especially pertaining to diseases of the nervous system and mental diseases. With regard to mortality, we found that FASD diagnosis is associated with increased risk of mortality associated with circulatory, digestive, respiratory, endocrine, and nervous-system diseases.

These results were somewhat expected as the association between prenatal alcohol exposure and increased immune-related risks have been proven in both animal and human studies over the last few years^42^. Maternal alcohol ingestion has been associated with a two- to threefold increase in the risk of premature delivery for women who drink heavily or binge drink during pregnancy^43^. Considering that premature delivery is linked to increased risk of respiratory infections, influenza, and sepsis^42^, the association between FASD onset and increased risk of various diseases is unsurprising.

Like previous investigations, there was a great emphasis on the diagnosis of ‘mental and behavioral disorders’ (ICD-10 codes: F00-F99)^9^ among FASD patients and, in our population group, increased hospitalization and mortality risk were also associated with mental diseases.

However, surprisingly, deaths from external factors such as suicides, accidents, and poisoning by illegal drugs or alcohol were not associated with FASD diagnosis to a statistically significant degree in our study. Previous studies that have attributed mortality causes to the external, including suicides (15%) and accidents (14%) have proportionate increases, but more research must be done on associations between FASDs and these causes^23^. Internally, diseases of the nervous and respiratory systems (8%), digestive system (7%), congenital malformations (7%), mental and behavioral disorders (4%), and diseases of the circulatory system (4%)^23^, were mostly in alignment with the proportion of deaths in our investigation. In our study, diseases of the circulatory system, digestive system, and circulatory system were also one of the most common causes of death for FASD populations.

On the contrary, 3.0% of FASD deaths in our investigation were associated with various cancers, including digestive, respiratory, genital/urinary, and skin/bone/breast, which were not categorized in Than & Johnson’s investigation. It must be noted that in our study, suicide was not a major cause of mortality, which may be because our investigation looked at milder forms of FASDs compared to previous studies dealing with FAS, the most severe form of FASDs^23^.

In the case of circulatory diseases, cerebrovascular and ischemic conditions were associated with increased mortality risk among individuals with FASDs. This was in alignment with the existing body of literature that has noted that individuals with FAS may manifest structural alterations, such as neural tube defects and alterations in neocortical or cerebellar morphogenesis^5^. Because these alterations, especially with regard to cortical thickness, can indicate immature brain development and poorer general intellectual function ability, more in-depth studies are required regarding the association between FASD diagnosis and circulatory function^5^.

While women had increased hospitalization risk than men, men had increased all-cause mortality risk than women. In existing studies regarding hospitalizations in South Korea, it has been clearly stated that several factors, including socioeconomic and demographic status, are central to the crude rate of hospitalizations. In one study, it was found that risk of hospitalizations and mortality increased among low income groups, those residing in rural regions^44^. This was in alignment with our investigation where such individuals with lower SES were at increased risk of hospitalizations and death.

Of individuals with FASDs, we found that compared to their general population peers, those diagnosed with mood disorder due to known physiological condition, unspecified (F06.30), encephalopathy, and other and unspecified (static) (G93.4) had increased mortality risk.

While few studies have examined the types of FASDs in detail with regard to their association with cause-specific mortality, in one clinic-based study in the past, it was found that the rate of maternal mortality for children with static encephalopathy was 8.8%, highlighting the risk of FASDs not only on the diagnosed child, but the mother as well^45^. Encephalopathy diagnosis has also been associated with premature mortality among individuals with liver cirrhosis^46^, birth asphyxia and trauma, and infantile epilepsy, with early infantile epileptic encephalopathy syndrome (EIEE), also known as Ohtahara syndrome, causing deaths in 58.8% of those diagnosed within one year of seizure onset^47^.

Regarding the increased mortality risk of cancers, circulatory diseases, and respiratory diseases, more research regarding fetal/placental anomalies and their association with various carcinogens are warranted. Apropos of cancers, in one previous study of childhood cancer in children with various congenital anomalies, < 3% of children with anomalies were part of the fetal alcohol syndrome category, and thereby excluded from the final analyses^48^.

Significant associations with alcohol exposure have already been made with congenital structural heart defects like ventricular septal defects, atrial septal defects, and conotruncal defects, and clinicians have reported some cases of children with FASDs whom asymptomatic cardiac rhythm alterations were detected in absence of structural cardiovascular system anomalies during routine follow-up^49^.

As emphasized by Onesimo and colleagues and seen in the results of our investigation where diseases of the circulatory system were of great mortality risk to FASD populations, an alert must be made for clinicians, given the possibility of finding anomalies of heart conduction and rhythm in children affected by FASDs even without structural congenital heart disease^49^.

For clinicians, a thorough understanding of potential risk and protective factors may help in determining the ways in which to help with prevention and management of FASDs. As listed in the existing body of literature, risk factors associated with adverse life outcomes have ranged from the environmental (being over 12 years of age at FAS/FAE (fetal alcohol effects) diagnosis, having a ‘low’ percent of life in a stable/nurturing home, having an IQ ≥ 70, being a victim of physical, sexual abuse/domestic violence, having a ‘high’ percentage of life with another person abusing alcohol and/or drugs, and having a ‘high’ percentage of life where basic needs are not met) to sociodemographic (being male, suffering from FAE)^25^.

On the contrary, strong protective factors against various secondary disabilities like alcohol/drug problems and confinement have included living a high percent of one’s life in a stable/nurturing home, being diagnosed at a younger age, and having a diagnosis of FAS^25^.

Overall, any alcohol consumption has consequences on craniofacial development, supporting advice that complete abstinence from alcohol during pregnancy is the safest option^35^. Women must be advised to abstain from alcohol during pregnancy, even when drinking below the low-risk guidelines of their general population counterparts. Equally, women with a past history of alcohol and/or substance abuse must also be provided with appropriate interventions to prevent continued drinking by healthcare professionals, if planning on pregnancy and/or childbirth.

Physicians and nurses are in an ideal position to advise against alcohol consumption during pregnancy, and inform a woman who is drinking early in her pregnancy about the injuries her alcohol consumption brings about to fetal cells, organs, and limbs^50^. As more than one-half of all pregnancies in the United States are unintended, women who have the potential to conceive should also be educated about the potential risks of frequent drinking^50^. Primary care providers can also help those diagnosed with FAS or FASDs by informing them about the specific diseases, especially with regard to diseases of the nervous system and mental systems, for example that increase hospitalization and mortality risk.

Policymakers should also be aware that the lowering of alcohol taxes and relative reductions in the price of alcohol can result in an increase in alcohol-related hospitalizations, including maternal care for (suspected) fetal damage^51^.

Although no cures currently exist for the treatment of FASDs, certain intervention services that may help, as recommended by certain medical centers, are as follows: (1) a team that includes a special education teacher, a speech therapist, physical and occupational therapists, and a psychologist; (2) early intervention to help with walking, talking and social skills; (3) special services in school to help with learning and behavioral issues; (4) medications to help with some symptoms; (5) medical care for health problems, such as vision problems or heart abnormalities; (6) addressing alcohol and other substance use problems, if needed; (7) vocational and life skills training; and (8) counseling to benefit parents and the family in dealing with a child's behavioral problems.

## Conclusion

The current study supplements the body of evidence regarding the lifelong adverse effects of prenatal alcohol exposure on those diagnosed with FASDs. By systematically analyzing the hospitalizations and mortality causes of those diagnosed, in comparison with their general population peers, vulnerable sociodemographic and health-related characteristics were identified.

Although there are numerous limitations regarding our data and analogy, we believe that this study can have meaningful impact socially, politically, and economically, by raising awareness regarding the harms of FASDs and the risks involved with diagnosis. For South Korea, our study shows that the impact of FASDs affect a large proportion of hospitalizations and mortality. In our study population, around 20.52% of hospitalizations and 21.35% of mortalities were attributable to FASDs. While this estimate may not be an accurate proportion of the effects of FASDs for the entire South Korean population as we had a limited number of random controls, such figures show the need for more research regarding the impact that this disease has for hospitalizations and deaths in different countries. Future research that incorporates international collaboration with countries where FASD research is relatively more active (e.g. Canada, United States, United Kingdom, Australia etc.) would refine the variability of our estimates.

Considering the occurrence of lifelong adverse outcomes associated with hospitalizations and mortality, clinicians and policymakers should make sure to emphasize to potential mothers the importance of temperance. This is particularly the case with certain diseases which showed a sharp increase in hospitalization and mortality risk among individuals with FASDs compared to the general population, such as diseases of the nervous system, mental diseases, and diseases of the digestive system, circulatory system, respiratory system, and endocrine system.

It is also recommended that clinicians and policymakers invest in more research, especially with regard to cases of encephalopathy, disorders of the central nervous system, and mood disorders due to known physiological conditions which were FASD domains that showed an increase in hospitalization and mortality risk.

Finally, diagnostic clinics that provide for check-ups regarding circulatory and respiratory diseases, as well as provide funding for prevention programs (education, alcohol abuse treatment, advocacy, birth control) and long-term residential/job training programs/medical check-ups for individuals diagnosed with FASDs are necessary for the prevention and management of excessive hospitalizations and mortality.

## Supplementary information

Supplementary Information.
