# Supplementary material for: Hospitalizations and mortality among patients with fetal alcohol spectrum disorders: a prospective study
Source: Sci Rep. 2020 Nov 11;10:19512. doi: 10.1038/s41598-020-76406-6 (PMC7658994; doi:10.1038/s41598-020-76406-6)
Supplement: Supplementary file 1 — Supplementary Information. [file 41598_2020_76406_MOESM1_ESM.docx]

| **Supplementary 1. Results of Cox Model for FASD and Hospitalizations with Comparison of Varying Time Scales** | | | | | | | | | | |
| --- | --- | --- | --- | --- | --- | --- | --- | --- | --- | --- |
|  | **Hospitalization** | | | | | | | | | |
|  | **Time-on Study with Follow-up as Time Scale** | | | | | **Left Truncated Cohort with Attained Age as Time Scale** | | | | |
|  | **HR** | **95% CI** | | | ***p*-value** | **HR** | **95% CI** | | | ***p*-value** |
|  |  | **Lower** |  | **Upper** |  |  | **Lower** |  | **Upper** |  |
| **Fetal Alcohol Spectrum Disorder** |  |  |  |  |  |  |  |  |  |  |
| None | 1.00 | - |  | - |  | 1.00 | - |  | - |  |
| Fetal Alcohol Spectrum Disorder | 1.28 | 1.10 | - | 1.48 | 0.0014 | 1.25 | 1.05 | - | 1.49 | 0.0114 |
| **Sex** |  |  |  |  |  |  |  |  |  |  |
| Male | 1.00 | - |  | - |  | 1.00 | - |  | - |  |
| Female | 0.96 | 0.88 | - | 1.05 | 0.3894 | 1.01 | 0.93 | - | 1.11 | 0.7905 |
| **Age at Cohort Entry** |  |  |  |  |  |  |  |  |  |  |
| <20 | 0.78 | 0.67 | - | 0.90 | 0.0012 |  |  |  |  |  |
| 20-29 | 0.83 | 0.71 | - | 0.98 | 0.0300 |  |  |  |  |  |
| 30-39 | 0.80 | 0.68 | - | 0.93 | 0.0042 |  |  |  |  |  |
| 40-49 | 1.00 | - |  | - |  |  |  |  |  |  |
| 50-59 | 0.92 | 0.78 | - | 1.08 | 0.3043 |  |  |  |  |  |
| 60-69 | 1.15 | 0.98 | - | 1.35 | 0.0988 |  |  |  |  |  |
| ≥70 | 2.12 | 1.82 | - | 2.47 | <.0001 |  |  |  |  |  |
| **Income** |  |  |  |  |  |  |  |  |  |  |
| None | 1.04 | 0.80 | - | 1.36 | 0.7481 | 1.15 | 0.86 | - | 1.53 | 0.3392 |
| Low | 1.00 | 0.86 | - | 1.18 | 0.9575 | 1.01 | 0.86 | - | 1.19 | 0.8948 |
| Medium-low | 1.08 | 0.94 | - | 1.25 | 0.2730 | 1.08 | 0.93 | - | 1.26 | 0.3094 |
| Medium | 1.00 | - |  | - |  | 1.00 | - |  | - |  |
| Medium-high | 1.05 | 0.92 | - | 1.20 | 0.4381 | 1.04 | 0.91 | - | 1.20 | 0.5428 |
| High | 0.99 | 0.87 | - | 1.13 | 0.8898 | 1.00 | 0.88 | - | 1.15 | 0.9822 |
| **Medical Insurance** |  |  |  |  |  |  |  |  |  |  |
| Medical Aid | - |  |  |  |  | - |  |  |  |  |
| Insurance (Regional) | 1.13 | 1.04 | - | 1.24 | 0.0047 | 1.12 | 1.02 | - | 1.22 | 0.0157 |
| Insurance (Corporate) | 1.00 | - |  | - |  | 1.00 | - |  | - |  |
| **Disability Type** |  |  |  |  |  |  |  |  |  |  |
| None | 1.00 | - |  | - |  | 1.00 | - |  | - |  |
| Mental | 1.60 | 1.29 | - | 1.97 | <.0001 | 1.44 | 1.14 | - | 1.82 | 0.0020 |
| Sensory | 1.00 | 0.66 | - | 1.52 | 0.9986 | 1.23 | 0.81 | - | 1.86 | 0.3322 |
| Intellectual | 1.85 | 1.09 | - | 3.13 | 0.0227 | 2.20 | 1.23 | - | 3.95 | 0.0081 |
| Other* | 4.43 | 3.19 | - | 6.16 | <.0001 | 2.19 | 1.35 | - | 3.56 | 0.0016 |
| **Disability Severity** |  |  |  |  |  |  |  |  |  |  |
| No Disability | 1.00 | - |  | - |  | 1.00 | - |  | - |  |
| Level 1-2 | 1.73 | 1.34 | - | 2.24 | <.0001 | 1.24 | 0.91 | - | 1.70 | 0.1797 |
| Level 3-6 | - |  |  |  |  | - |  |  |  |  |
| **Charlson Comorbidity Index** |  |  |  |  |  |  |  |  |  |  |
| None | 1.00 | - |  | - |  | 1.00 | - |  | - |  |
| One | 1.54 | 1.32 | - | 1.81 | <.0001 | 1.42 | 1.19 | - | 1.68 | <.0001 |
| Two | 1.82 | 1.53 | - | 2.17 | <.0001 | 1.84 | 1.53 | - | 2.20 | <.0001 |
| ≥ Three | 2.74 | 2.37 | - | 3.18 | <.0001 | 2.75 | 2.36 | - | 3.21 | <.0001 |
| **Region** |  |  |  |  |  |  |  |  |  |  |
| Metropolitan | 1.00 | - |  | - |  | 1.00 | - |  | - |  |
| City | 1.50 | 1.34 | - | 1.69 | <.0001 | 1.58 | 1.40 | - | 1.78 | <.0001 |
| Other | 2.26 | 2.04 | - | 2.50 | <.0001 | 2.41 | 2.17 | - | 2.68 | <.0001 |
| **Year of Cohort Entry** |  |  |  |  |  |  |  |  |  |  |
| 2003 | 1.00 | - |  | - |  | 1.00 | - |  | - |  |
| 2004 | 1.83 | 1.59 | - | 2.12 | <.0001 | 1.57 | 1.33 | - | 1.84 | <.0001 |
| 2005 | 2.91 | 2.42 | - | 3.52 | <.0001 | 2.52 | 2.05 | - | 3.11 | <.0001 |
| 2006 | 3.48 | 2.75 | - | 4.41 | <.0001 | 2.94 | 2.23 | - | 3.86 | <.0001 |
| 2007 | 7.43 | 5.93 | - | 9.32 | <.0001 | 5.97 | 4.55 | - | 7.82 | <.0001 |
| 2008 | 7.76 | 6.16 | - | 9.76 | <.0001 | 7.77 | 5.95 | - | 10.16 | <.0001 |
| 2009 | 7.68 | 5.80 | - | 10.16 | <.0001 | 8.33 | 5.96 | - | 11.64 | <.0001 |
| 2010 | 7.24 | 5.27 | - | 9.96 | <.0001 | 16.72 | 12.09 | - | 23.12 | <.0001 |
| 2011 | 4.64 | 3.01 | - | 7.16 | <.0001 | 19.84 | 13.60 | - | 28.93 | <.0001 |
| 2012 | 2.50 | 1.25 | - | 5.01 | 0.0098 | 16.42 | 9.26 | - | 29.13 | <.0001 |
| 2013 | - |  |  |  |  | - |  |  |  |  |
| **intellectual, kidney, musculoskeletal, circulatory, respiratory, nervous system, developmental etc.* | | | | | | | | | | |

| **Supplementary 2. Results of Cox Model for FASD and Mortality with Comparison of Varying Time Scales** | | | | | | | | | | |
| --- | --- | --- | --- | --- | --- | --- | --- | --- | --- | --- |
|  | **Mortality** | | | | | | | | | |
|  | **Time-on Study with Follow-up as Time Scale** | | | | | **Left Truncated Cohort with Attained Age as Time Scale** | | | | |
|  | **HR** | **95% CI** | | | ***p*-value** | **HR** | **95% CI** | | | ***p*-value** |
|  |  | **Lower** |  | **Upper** |  |  | **Lower** |  | **Upper** |  |
| **Fetal Alcohol Spectrum Disorder** |  |  |  |  |  |  |  |  |  |  |
| None | 1.00 | - |  | - |  | 1.00 | - |  | - |  |
| Fetal Alcohol Spectrum Disorder | 1.44 | 1.17 | - | 1.77 | 0.0005 | 1.33 | 1.07 | - | 1.67 | 0.0118 |
| **Sex** |  |  |  |  |  |  |  |  |  |  |
| Male | 1.00 | - |  | - |  | 1.00 | - |  | - |  |
| Female | 0.62 | 0.55 | - | 0.70 | <.0001 | 0.60 | 0.52 | - | 0.68 | <.0001 |
| **Age at Cohort Entry** |  |  |  |  |  |  |  |  |  |  |
| <20 | 0.05 | 0.03 | - | 0.11 | <.0001 |  |  |  |  |  |
| 20-29 | 0.14 | 0.07 | - | 0.26 | <.0001 |  |  |  |  |  |
| 30-39 | 0.30 | 0.19 | - | 0.48 | <.0001 |  |  |  |  |  |
| 40-49 | 1.00 | - |  | - |  |  |  |  |  |  |
| 50-59 | 2.79 | 2.07 | - | 3.74 | <.0001 |  |  |  |  |  |
| 60-69 | 6.42 | 4.87 | - | 8.46 | <.0001 |  |  |  |  |  |
| ≥70 | 31.63 | 24.45 | - | 40.91 | <.0001 |  |  |  |  |  |
| **Income** |  |  |  |  |  |  |  |  |  |  |
| None | 1.36 | 1.02 | - | 1.80 | 0.0335 | 1.57 | 1.15 | - | 2.12 | 0.0040 |
| Low | 0.74 | 0.59 | - | 0.94 | 0.0121 | 0.68 | 0.53 | - | 0.88 | 0.0033 |
| Medium-low | 0.96 | 0.77 | - | 1.20 | 0.7312 | 0.84 | 0.66 | - | 1.08 | 0.1722 |
| Medium | 1.00 | - |  | - |  | 1.00 | - |  | - |  |
| Medium-high | 0.74 | 0.60 | - | 0.91 | 0.0038 | 0.73 | 0.58 | - | 0.92 | 0.0066 |
| High | 0.75 | 0.62 | - | 0.90 | 0.0021 | 0.72 | 0.58 | - | 0.88 | 0.0018 |
| **Medical Insurance** |  |  |  |  |  |  |  |  |  |  |
| Medical Aid | - |  |  |  |  | - |  |  |  |  |
| Insurance (Regional) | 0.83 | 0.73 | - | 0.95 | 0.0060 | 0.94 | 0.81 | - | 1.09 | 0.3999 |
| Insurance (Corporate) | 1.00 | - |  | - |  | 1.00 | - |  | - |  |
| **Disability Type** |  |  |  |  |  |  |  |  |  |  |
| None | 1.00 | - |  | - |  | 1.00 | - |  | - |  |
| Mental | 1.88 | 1.52 | - | 2.33 | <.0001 | 1.93 | 1.53 | - | 2.42 | <.0001 |
| Sensory | 1.47 | 1.07 | - | 2.03 | 0.0181 | 1.15 | 0.80 | - | 1.65 | 0.4668 |
| Intellectual | 4.21 | 2.27 | - | 7.81 | <.0001 | 3.77 | 1.85 | - | 7.69 | 0.0003 |
| Other* | 4.02 | 2.93 | - | 5.53 | <.0001 | 2.63 | 1.84 | - | 3.75 | <.0001 |
| **Disability Severity** |  |  |  |  |  |  |  |  |  |  |
| No Disability | 1.00 | - |  | - |  | 1.00 | - |  | - |  |
| Level 1-2 | 1.62 | 1.27 | - | 2.07 | 0.0001 | 1.81 | 1.39 | - | 2.36 | <.0001 |
| Level 3-6 | - |  |  |  |  | - |  |  |  |  |
| **Charlson Comorbidity Index** |  |  |  |  |  |  |  |  |  |  |
| None | 1.00 | - |  | - |  | 1.00 | - |  | - |  |
| One | 0.73 | 0.55 | - | 0.95 | 0.0204 | 0.69 | 0.50 | - | 0.94 | 0.0197 |
| Two | 0.72 | 0.55 | - | 0.93 | 0.0115 | 0.84 | 0.63 | - | 1.13 | 0.2552 |
| ≥ Three | 0.58 | 0.46 | - | 0.72 | <.0001 | 0.73 | 0.57 | - | 0.94 | 0.0145 |
| **Region** |  |  |  |  |  |  |  |  |  |  |
| Metropolitan | 1.00 | - |  | - |  | 1.00 | - |  | - |  |
| City | 0.98 | 0.83 | - | 1.14 | 0.7522 | 1.01 | 0.85 | - | 1.21 | 0.9054 |
| Other | 0.96 | 0.84 | - | 1.10 | 0.5787 | 1.02 | 0.87 | - | 1.18 | 0.8263 |
| **Year of Cohort Entry** |  |  |  |  |  |  |  |  |  |  |
| 2003 | 1.00 | - |  | - |  | 1.00 | - |  | - |  |
| 2004 | 1.13 | 0.89 | - | 1.45 | 0.3210 | 1.23 | 0.95 | - | 1.59 | 0.1236 |
| 2005 | 1.35 | 1.01 | - | 1.81 | 0.0463 | 1.43 | 1.04 | - | 1.97 | 0.0259 |
| 2006 | 1.57 | 1.13 | - | 2.17 | 0.0071 | 1.62 | 1.12 | - | 2.35 | 0.0105 |
| 2007 | 1.44 | 1.04 | - | 2.01 | 0.0303 | 1.53 | 1.05 | - | 2.21 | 0.0265 |
| 2008 | 1.80 | 1.35 | - | 2.41 | <.0001 | 2.11 | 1.53 | - | 2.92 | <.0001 |
| 2009 | 1.75 | 1.19 | - | 2.57 | 0.0046 | 2.35 | 1.54 | - | 3.57 | <.0001 |
| 2010 | 2.26 | 1.56 | - | 3.26 | <.0001 | 2.62 | 1.71 | - | 4.03 | <.0001 |
| 2011 | 1.92 | 1.28 | - | 2.88 | 0.0017 | 1.86 | 1.12 | - | 3.09 | 0.0171 |
| 2012 | 2.77 | 1.77 | - | 4.34 | <.0001 | 2.70 | 1.35 | - | 5.39 | 0.0050 |
| 2013 | 2.83 | 1.45 |  | 5.51 | 0.0022 |  |  |  |  |  |
| **intellectual, kidney, musculoskeletal, circulatory, respiratory, nervous system, developmental etc.* | | | | | | | | | | |
